# Evaluating the inhibitory effect of resveratrol on the multiplication of several *Babesia* species and *Theileria equi* on *in vitro* cultures, and *Babesia microti* in mice

**DOI:** 10.3389/fphar.2023.1192999

**Published:** 2023-05-30

**Authors:** Shimaa Abd El-Salam El-Sayed, El-Sayed El-Alfy, Mohamed Z. Sayed-Ahmed, Uday Kumar Mohanta, Saad S. Alqahtani, Nawazish Alam, Sarfaraz Ahmad, Md Sajid Ali, Ikuo Igarashi, Mohamed Abdo Rizk

**Affiliations:** ^1^ National Research Center for Protozoan Diseases, Obihiro University of Agriculture and Veterinary Medicine, Obihiro, Hokkaido, Japan; ^2^ Department of Biochemistry and Chemistry of Nutrition, Faculty of Veterinary Medicine, Mansoura University, Mansoura, Egypt; ^3^ Parasitology Department, Faculty of Veterinary Medicine, Mansoura University, Mansoura, Egypt; ^4^ Department of Clinical Pharmacy, College of Pharmacy, Jazan University, Jizan, Saudi Arabia; ^5^ Department of Clinical Pharmacy, College of Pharmacy, King Khalid University, Abha, Saudi Arabia; ^6^ Department of Pharmaceutics, College of Pharmacy, Jazan University, Jizan, Saudi Arabia; ^7^ Department of Internal Medicine and Infectious Diseases, Faculty of Veterinary Medicine, Mansoura University, Mansoura, Egypt

**Keywords:** resveratrol, diminazene aceturate, imidocarb dipropionate, azithromycin, babesia, *T. equi*, cardiac troponin T

## Abstract

**Introduction:** Histone post-translational modification is one of the most studied factors influencing epigenetic regulation of protozoan parasite gene expression, which is mediated by histone deacetylases (KDACs) and acetyltransferases (KATs).

**Objective and methods:** The present study investigated the role of resveratrol (RVT) as an activator of histone deacetylases in the control of various pathogenic Babesia sp. and Theileria equi *in vitro*, as well as *B. microti* infected mice *in vivo* using fluorescence assay. Its role in mitigating the side effects associated with the widely used antibabesial drugs diminazene aceturate (DA) and azithromycin (AZM) has also been investigated.

**Results:** The *in vitro* growth of *B. bovis*, *B. bigemina*, *B. divergens*, *B. caballi* and Theileria equi (*T. equi*) was significantly inhibited (P < 0.05) by RVT treatments. The estimated IC50 values revealed that RVT has the greatest inhibitory effects on *B. bovis* growth *in vitro*, with an IC50 value of 29.51 ± 2.46 µM. Reverse transcription PCR assay showed that such inhibitory activity might be attributed to resveratrol’s stimulatory effect on *B. bovis* KDAC3 *(BbKADC3)* as well as its inhibitory effect on BbKATS. RVT causes a significant decrease (P < 0.05) in cardiac troponin T (cTnT) levels in heart tissue of *B. microti*- infected mice, thereby indicating that RVT may play a part in reducing the cardiotoxic effects of AZM. Resveratrol showed an additive effect with imidocarb dipropionate *in vivo*. Treatment of *B. microti*-infected mice with a combined 5 mg/kg RVT and 8.5 mg/kg ID resulted in an 81.55% inhibition at day 10 postinoculation (peak of parasitemia).

**Conclusion:** Our data show that RVT is a promising antibabesial pharmacological candidate with therapeutic activities that could overcome the side effects of the currently used anti-Babesia medications.

## 1 Introduction

Changes in chromatin structure that affect parasite virulence, differentiation and cell cycle control are most frequently attributed to epigenetic gene regulation (Gibney and Nolan, 2010). The complicated life cycle of Babesia parasites in various hosts requires rapid alteration of the organism’s gene expression profile to start extensive developmental programs in response to environmental cues including stress, switching between hosts or host defenses (Elsworth and Duraisingh, 2021). However, despite increasing the importance of the epigenetic control of gene expression to understanding parasite biology, the mechanisms involved in this regulation are only partially characterised. For protozoan parasites such as Babesia, the parasite exhibits distinctive and diverse mechanisms of epigenetic gene control, even though various fundamental principles of epigenetic gene regulation are comparable to those of mammalian cells and model systems (Brownell et al., 1996). The most common epigenetic modifications occur on DNA or the associated proteins, most notably histones, which can be post-translationally modified by acetylation yielding euchromatin (loose, accessible) or deacetylated yielding heterochromatin (compact, inaccessible) based on gene activation or repression (Croken et al., 2012). These modifications are regulated by histone acetyltransferases (KATs) and deacetylases (KDACs) (Croken et al., 2012). Histone acetyltransferases are epigenetic enzymes that add acetyl groups to lysine residues of cellular proteins such as histones, transcription factors, nuclear receptors and enzymes (Wapenaar and Dekker, 2016). Meanwhile, KDACs are a class of enzymes that remove acetyl groups from an ε-N-acetyl lysine amino acid on a histone, thereby allowing the histones to wrap the DNA more tightly and both were conserved by malaria parasites and the piroplasm (Wapenaar and Dekker, 2016). Given the significance of epigenetics in parasite biology, the mechanism of parasite epigenetics can open up a new field of research for the development of novel therapies for protozoan diseases (Croken et al., 2012; Vanagas et al., 2012). Over the last few decades, epigenetic regulation of gene expression has been extensively studied, particularly concerning human disease. It has further been demonstrated that it can serve an important role in apicomplexan parasites via histone modification (Sindikubwabo et al., 2017). Histone deacetylase (KDAC) and histone methyltransferases inhibitors are a promising targets for the development of novel anti-babesial drugs (Munkhjargal et al., 2012; Gurboga et al., 2021). Noteworthy, apicidin, a histone deacetylase inhibitor, inhibited *B. bovis, B. bigemina*, and *B. Microti* in mice (Munkhjargal, 2009), and furamidine, a histone methyltransferase inhibitor, inhibited B. Xinjiang *in vitro* (Li X et al., 2022). However, there is no evidence that KDACs activators and KATs antagonists can be used concurrently.

Resveratrol (RVT, 3,5,4′-trihydroxystilbene) is a natural polyphenol compound that was initially isolated from the roots of the white hellebore Veratrum grandiflorum O. Loes in 1939 by Michio Takaoka (Takaoka, 1939). Currently, this natural product can be found in various plants, particularly grape species, in response to fungal infections and ultraviolet radiation (Langcake et al., 1979). Notably, RVT is one of the main natural compounds studied worldwide because of its potential therapeutic use in the treatment of various diseases, including cancer, diabetes, cardiovascular diseases, neurodegenerative diseases and metabolic disorders (Bhat et al., 2001; Aggarwal et al., 2004; Duarte et al., 2015; Bostanghadiri et al., 2017).

Resveratrol has anti-protozoal activity against Leishmania sp. (Ferreira et al., 2014; Mousavi et al., 2022), *Trypanosoma cruzi* (*T. cruzi*) (Campo, 2017; Rodriguez et al., 2022), *Entamoeba histolytica* (Pais-Morales et al., 2016), and Toxoplasma gondii (Chen et al., 2019; Contreras et al., 2021). However, the inhibitory efficacy of RVT against *Babesia* and Theileria was not yet evaluated. Therefore, in the present study, we evaluated the inhibitory efficacy of RVT against the growth of bovine *Babesia* and equine piroplasm *in vitro* and against the growth of *B. Microti* in mice when used as a monotherapy or in combination with three traditional antibabesial drugs; diminazene aceturate (DA), imidocarb dipropionate (ID) and azithromycin (AZM). Furthermore, we evaluated the epigenetic regulation of RVT on Babesia as a target to KATs and KDACs and detected the cardioprotective efficacy of RVT when used in combination with a drug widely used for the treatment of babesiosis and known by its side effect on the heart, AZM.

## 2 Materials and methods

### 2.1 Chemicals

SYBR Green I (SGI) nucleic acid stain (Lonza, Rockland, United States; 10,000 x) was stored at −20 °C and thawed before use for Babesia inhibition assay *in vitro* and *in vivo*. A lysis buffer was prepared in advance and stored at 4 °C. Resveratrol (Sigma-Aldrich, Japan) stock solution was dissolved in double distilled water (DDW) at 0.0022 g/100 μL and kept at −30°C until use. The commonly used antibabesial drugs DA (Ganaseg, Ciba-Geigy Japan Ltd., Tokyo, Japan) and ID (Sigma-Aldrich, Japan) were utilized as positive control drugs. Furthermore, the compound MMV396693 (MolPort, Latvia) and AZM (Tocris Bioscience, UK) were used for combination inhibition assays.

### 2.2 Parasites

Babesia bovis (Texas strain), *B. bigemina* (Argentina strain), *B. divergens* (German strain), and *B. caballi* and Theileria equi (USDA strain) were grown and maintained in purified bovine or equine red blood cells (RBCs) using a microaerophilic stationary-phase culture technique at 37°C under the environmental condition of 5% CO_2_, 5% O_2_ and 90% N2 as detailed in our previous studies (Rizk et al., 2015; Rizk et al., 2017b; Rizk et al., 2021, 2023).

### 2.3 *In vitro* growth inhibition assay and viability test

The inhibitory effect of RVT on the screened piroplasm growth was investigated using an SGI-based fluorescence assay (Rizk et al., 2015; Rizk et al., 2016; Rizk et al., 2020). Double 96-well plates (Nunc, Roskilde, Denmark) were used to cultivate bovine Babesia and equine Babesia/Theileria pRBCs with either media alone or medium with the appropriate doses of RVT ranging from 0.005 to 200 μM. DA was used as a positive control with concentrations ranging from 0.25 to 10 μM. Meanwhile, cultures without the drug and/or cultures containing only the used solvent DDW (0.02%) were used as negative controls. A viability assay was performed as previously detailed in our studies to assess the antipiroplasm efficacy of RVT following the stop of the treatment (Rizk et al., 2016; Rizk et al., 2017b; Rizk et al., 2020; Rizk et al., 2021).

A pharmaceutical combination experiment was carried out using the Chou-Talalay approach at a constant ratio (Rizk et al., 2023). A two-drug combination (RVT + DA, RVT + ID, RVT + MMV396693) was added in triplicate to the wells containing *B. bovis-, B. bigemina- and T. equi-infected* RBC in a 96-well plate at concentrations of 0.25 x IC50, 0.5 x IC50, IC50, 2 x IC50 and 4 x IC50.

All of the *in vitro* experiments were performed at 1% parasitemia, and 2.5% hematocrit (HCT) for *B. bovis* and *B. bigemina* and 5% HCT for other screened parasites (Rizk et al., 2017b; Rizk et al., 2020; Rizk et al., 2021). After 4 days of incubation, 100 μL a lysis buffer mixed with a 2× SGI was added directly to each well in the 96- wells plates. Then, the emitted fluorescence signals were determined using a fluorescence spectrophotometer. The experiments were repeated thrice.

### 2.4 Reverse transcription-PCR (RT-PCR)

Reverse transcription-PCR (RT-PCR) was performed to investigate the impact of RVT administration on BbKATs and BbKDAC3 mRNA transcription based on the previously described method (Aboulaila et al., 2012) with some modifications. Resveratrol was added to *B. bovis* in 24-well culture plates for 8 h at the 99% inhibitory concentration (IC99) (58.42 μM). The solvent served as a negative control. A commercial RNeasy minikit (QIAGEN) was used to extract total RNA from RBCs based on the manufacturer’s instructions. The extracted RNA (150 ng) from the treated- and controlled-cultures was used to amplify BbKATs and BbKDAC3 genes using a One Step RNA Kit (AMV) (Takara, Japan) following the manufacturer’s protocol. The *B. bovis* profilin (BbPROF) gene is used as the control gene. The used forward and reverse primers are listed in Table 1. The used PCR conditions were as follows: 30 min at 50°C for reverse transcription, denaturation at 94°C for 2 min then 30 cycles of denaturation at 94°C for 30 s, annealing at 55°C for 30 s for BbKDAC3, BbPROF and 57°C for BbKATs then, extension at 72°C for 1 min, and final extension at 72°C for 5 min. Finally, the amplified products were analysed by electrophoresis on 2.0% (w/v) agarose gels and visualised by a UV transilluminator after staining with ethidium bromide (Nippon Gene, Tokyo, Japan). The experiment was repeated thrice.

**TABLE 1:** Gene-specific primers for amplifying BbKATs, BbKDAC3, and BbPROF.

Gene oligonucleotide primer	Gene oligonucleotide primer	References
BbKATs	F 5′ -GAA​CGA​ACC​ATC​GCA​CAC​TA-3′	This study
	R 5′ -CGT​GGC​AGG​TAT​CCT​TTT​GT-3′	
BbKDAC3	F 5′ -ACG​AAT​TCA​TGG​AGA​AGA​GAG​TTT​CTT​A-3′ R 5′ - ACC​TCG​AGC​TAT​ATC​GGT​ATA​TGC​TGG​T-3′	Munkhjargal et al. (2012)
BbPROF	F 5′ - ACG​AAT​TCA​TGG​CAG​ATT​GGG​TTC-3′	Munkhjargal et al. (2012)
	R 5′ -ACC​TCG​AGT​TAA​TAA​CCA​TTG​GCA​GCC-3′	

### 2.5 Chemotherapeutic efficacy of RVT on the growth of *B. microti* in mice

The *in vivo* inhibitory effect of RVT on the growth of *B. Microti* (Munich strain) was evaluated twice in BALB/c mice aged 8 weeks (CLEA, Tokyo, Japan) using a fluorescence-based SGI assay (Rizk et al., 2017a; Rizk et al., 2020). All mice were housed in pathogen-free environments. A total of 50 female BALB/c mice were equally divided into 10 groups. All animals were intraperitoneally inoculated with 1 × 107 *B. Microti*-infected RBCs, except for the mice in the first group which remained uninfected and served as negative control. When the parasitemia in the infected mice reached 1%, mice in the experimental groups were treated with the specific drug. Each RVT, DA and ID was dissolved in DDW (12.5%) before inoculation. Meanwhile, AZM was dissolved in DMSO (0.02%).

As a positive placebo control, mice in the second group were given intraperitoneal doses of the used solvent. In addition, mice in the third group were given DA subcutaneously at 25 mg/kg. Meanwhile, mice in the fourth group were given 25 mg/kg AZM intraperitoneally. Moreover, mice in the fifth group were given 8.5 mg/kg ID. Furthermore, mice in the sixth group were intraperitoneally administered a non-toxic dose (5 mg/kg) from RVT. The remaining groups (7th and 8th) were injected with drug combinations of RVT 5 mg/kg + AZM 25 mg/kg or RVT 5 mg/kg + ID 8.5 mg/kg. All drugs were administrated for 5 successive days except those treated with AZM monotherapy or AZM combined with RVT, which were treated for 14 successive days.

A venous tail blood sample (2.5 μL) was drawn from each mouse every 48 h until 28 days post-inoculation or the end of parasitemia and placed in a 96-well plate with RPMI 1640 Medium previously mixed with 50 μL lysis buffer. Afterwards, each well is properly mixed with 50 μL of lysis buffer containing 2X SG I nucleic acid stain. Then, the plate was incubated in the dark for 1 h. Subsequently, the emitted fluorescence signals as indicators of the parasitemia were determined as previously detailed.

### 2.6 PCR detection of *B. microti* in mice

A nested PCR assay targeting the *B. Microti* small subunit rRNA (ss-rRNA) gene was performed to determine the presence of parasite nucleic acid remnant in the blood and tissue of the treated mice (Rizk et al., 2023). On day 28 post-infection, blood and tissue samples (heart, lung, liver, kidney and spleen) were collected from *B. Microti*–infected mice treated with 25 mg/kg DA, RVT 5 mg/kg + ID 8.5 mg/kg, the solvent (positive control) and non-treated mice (negative control). NucleoSpin tissue kit (Macherey-Nagel, Düren, Germany) and a QIAamp DNA Blood Mini Kit (Qiagen, Tokyo, Japan) were used for DNA extraction from tissues and blood, respectively. PCR cycling was carried out as previously detailed in our study (Rizk et al., 2017a). The experiment was repeated twice.

### 2.7 Cardiac troponin T (cTnT) level

For the cardioprotective effect of RVT to be determined, cardiac troponin T (cTnT) level in the plasma, and heart tissue of B. microti-infected mice treated with DA 25 mg/kg, AZM 25 mg/kg, AZM 25 mg/kg + RVT 5 mg/kg and RVT 5 mg/kg as well as in those treated with the solvent (positive control) and non-treated mice (negative control). The plasma was collected on day 0 (pre-infection), day 10 and day 28 post-infection. Meanwhile, heart tissue samples were collected on day 28 post-infection. Cardiac troponin T level was determined using commercially available mouse ELISA reagent kits (MyBioSource, Inc., United States) based on the manufacturer’s recommendations. The experiment was repeated twice.

### 2.8 Statistical analysis

The significant differences between the groups in the present study were determined using GraphPad Prism (version 5.0 for Windows; GraphPad Software, Inc., San Diego, CA, United States). In addition, a one-way ANOVA test was used and a *p*-value lower than 0.05 was considered statistically significant.

### 2.9 Ethics approval and consent to participate

All experimental protocols used in this study were authorized by the Obihiro University of Agriculture and Veterinary Medicine’s Animal Care and Use Committee (Approval No. 27-65). All research was done following the Fundamental Guidelines for the Proper Conduct of Animal Experiments and Related Activities at Academic Research Institutions published by the Ministry of Education, Culture, Sports, Science and Technology of Japan. The following were the IDs for the pathogen experiment: Babesia microti: 20170905; equine piroplasm parasites: 201910-2; and bovine Babesia: 201708-4.

## 3 Results

### 3.1 *B. bovis* exhibited the highest response to the inhibitory effect of RVT

**FIGURE 1:**
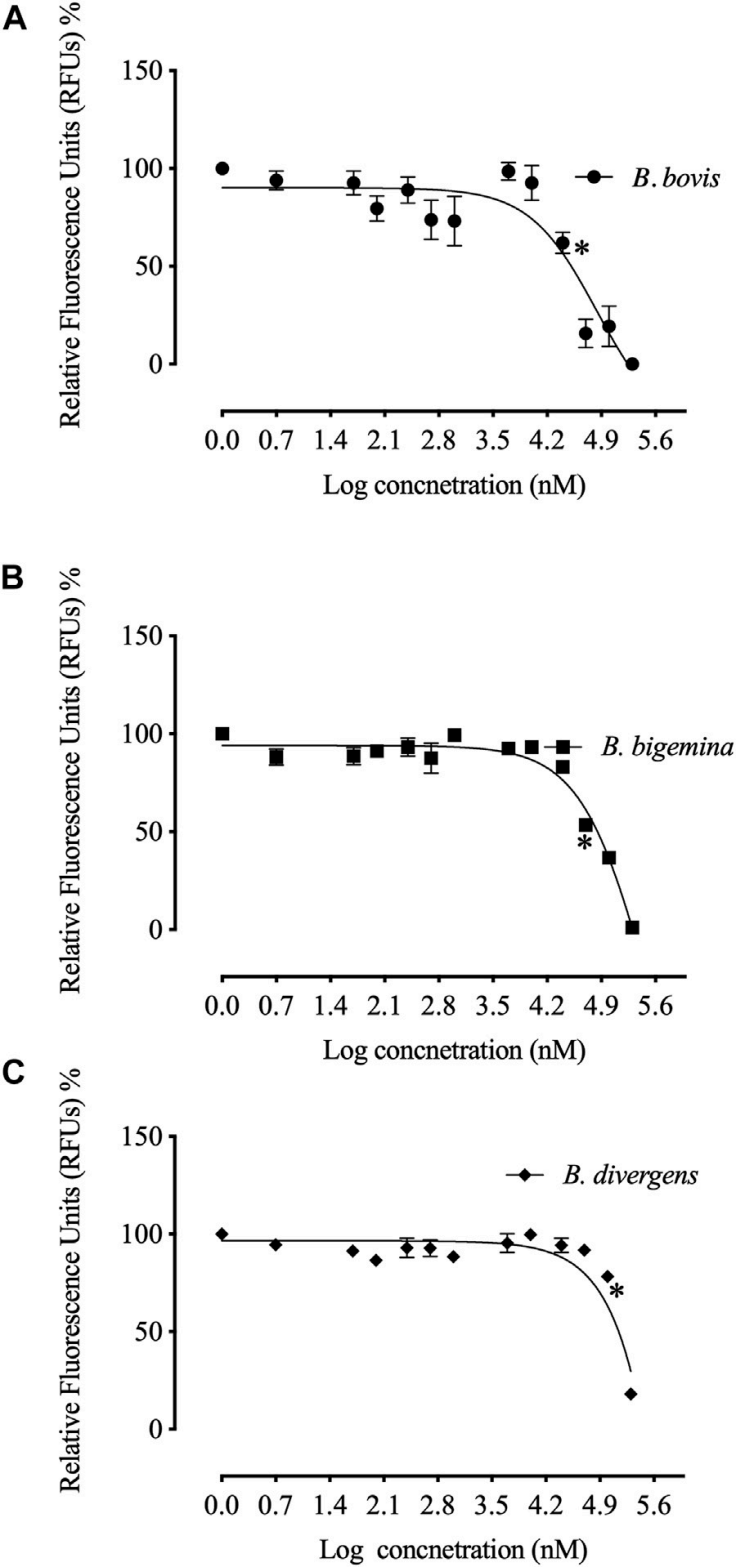
Correlation between relative fluorescence units (RFUs) and the log concentrations of resveratrol (nM) on bovine Babesia parasites *in vitro*. **(A)** *B. bovis*. **(B)**. *B. bigemina*. **(C)** *B. divergens*. Each value represents the mean of triplicate wells after subtraction of the background fluorescence for non-parasitised RBCs. Asterisks indicate a significant difference (ANOVA; *p < 0.05) between the resveratrol-treated and the control cultures.

**FIGURE 2:**
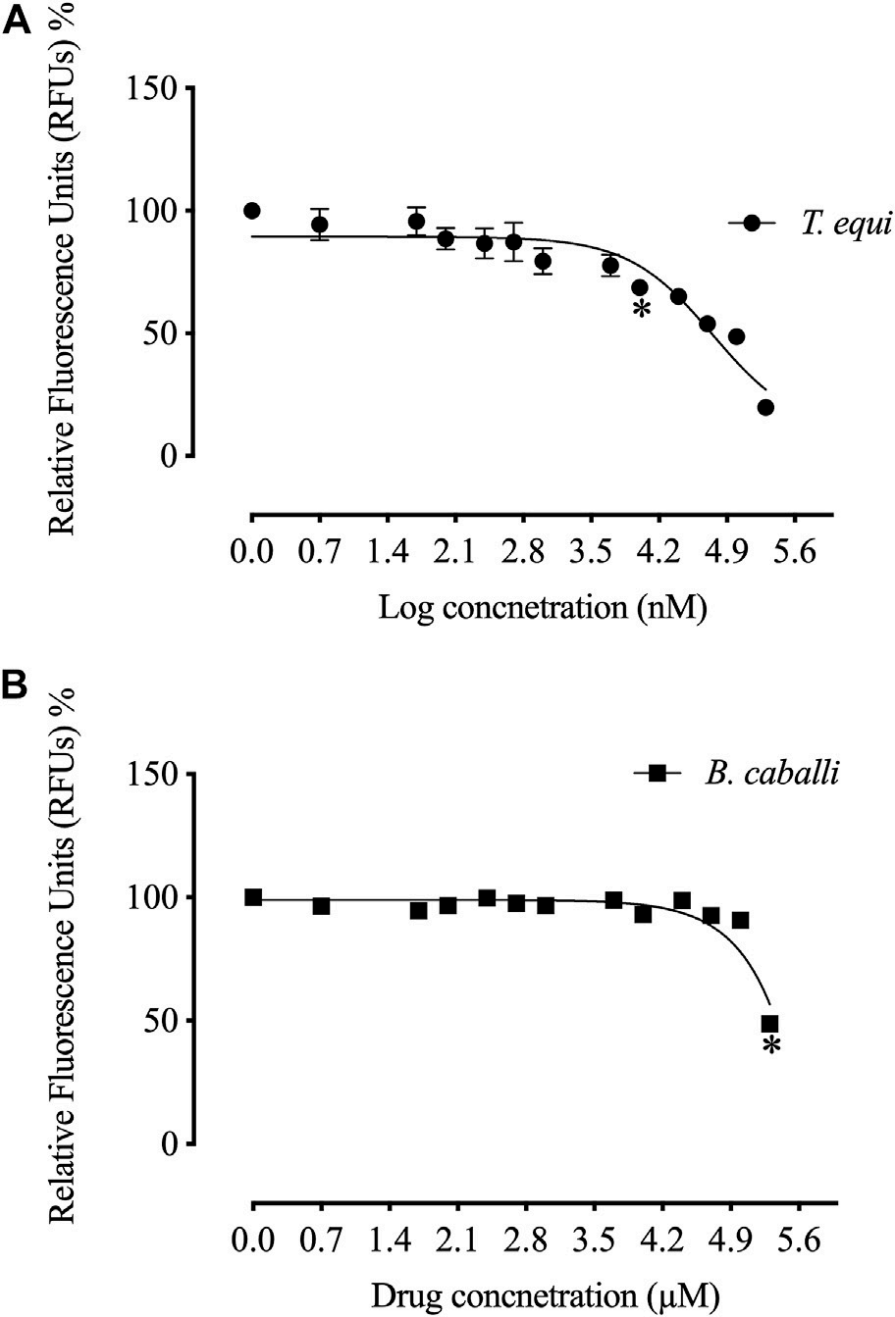
Correlation between relative fluorescence units (RFUs) and the log concentrations of resveratrol (nM) on equine piroplasm *in vitro*. **(A)** *T. equi*. **(B)** *B. caballi*. Each value represents the mean of triplicate wells after subtraction of the background fluorescence for non-parasitised RBCs. Asterisks indicate a significant difference (ANOVA; *p < 0.05) between the resveratrol-treated and the control cultures.

The *in vitro* growth of *Babesia bovis*, *B. bigemina* and *B. divergens* was significantly inhibited (*p* < 0.05) by RVT treatments of 25 μM, 50 μM and 100 μM, respectively (Figure 1). The estimated IC50 values revealed that RVT has the greatest inhibitory effects on *B. bovis* growth (Table 2). The *in vitro* growth of Theileria equi and *B. caballi* was significantly inhibited (*p* < 0.05) by RVT treatments of 10 μM and 200 μM, respectively (Figure 2).

**TABLE 2:** IC50 values of resveratrol and diminazene aceturate evaluated for bovine Babesia and equine Babesia and Theileria parasites.

Organism	IC50 (µM)^a^	
	Resveratrol	Diminazene aceturate
B. bovis	29.51 ± 2.46	0.75 ± 0.08
B. bigemina	56.25 ± 8.83	1.24 ± 0.007
B. divergens	148.67 ± 5.88	0.41 ± 0.06
T. equi	53.57 ± 5.04	0.82 ± 0.05
B. caballi	185.75 ± 2.74	0.13 ± 0.004

^a^ Each drug concentration was made in triplicate in each experiment, and the final obtained IC50 represents the mean and standard deviation of three separate experiments.

The calculated IC50 values of RVT treatments on all screened parasites were higher than those of the commonly used antibabesial drug, DA (Table 2). With 50 μM of RVT, subsequent viability tests revealed that there was no regrowth of *B. bovis* (Supplementary Table S1). These results indicate the higher *in vitro* efficacy of RVT on *B. bovis* growth inhibition rather than other bovine and equine piroplasms. However, none of the other piroplasmids displayed regrowth at a concentration of 200 μM (Supplementary Table S1). Thus, RVT was used combined with either DA, ID or MMV396693 to treat the parasites whose growth exhibited a high inhibition in the presence of RVT monotherapy; *B. bovis*, *B. bigemina*, and *T. equi*.

All two-drug combinations RVT + DA, RVT + ID or RVT + MMV396693 showed an antagonist effect on the growth of all selected parasites (Table 3).

**TABLE 3:** Combination index (CI) value of a two-drug combination between resveratrol, and other antibabesial drugs on the *in vitro* growth of *B. bovis*, *B. bigemina*, and *T. equi*.

Drug combinations^a^	CI value at				Weighted average CI values^b^	Degree of synergism^c^
	IC50	IC75	IC90	IC95		
Diminazene aceturate						
B. bovis	>10	>10	>10	10.21	>10	Antagonism
B. bigemina	13.12	10.29	8.08	6.86	9.58	Antagonism
T. equi	>10	>10	7.67	3.13	>10	Antagonism
Imidocarb dipropionate						
B. bovis	>10	>10	>10	>10	>10	Antagonism
B. bigemina	>10	>10	>10	>10	>10	Antagonism
T. equi	0.11	0.24	14.62	8.74	5.92	Antagonism
MMV396693						
B. bovis	>10	>10	>10	>10	>10	Antagonism
B. bigemina	>10	>10	>10	>10	>10	Antagonism
T. equi	>10	>10	>10	>10	>10	Antagonism

CI, value, combination index value; IC50, 50% inhibition concentration.

^a^ Two-drug combination between resveratrol, and other antibabesial drugs at a concentration of approximately 0.25 x IC50, 0.5 x IC50, IC50, 2 x IC50 and 4 x IC50 (constant ratio).

^b^ The higher inhibition is preferable; thus, the weighted average CI, value was calculated with the formula [(1 x IC50) + (2 x IC75) + (3 x IC90) + (4 x IC95)]/10.

^c^ The degree of synergism was determined based on the Chou-Talalay method.

### 3.2 RVT decreased the mRNA transcription of BbKATs

**FIGURE 3:**
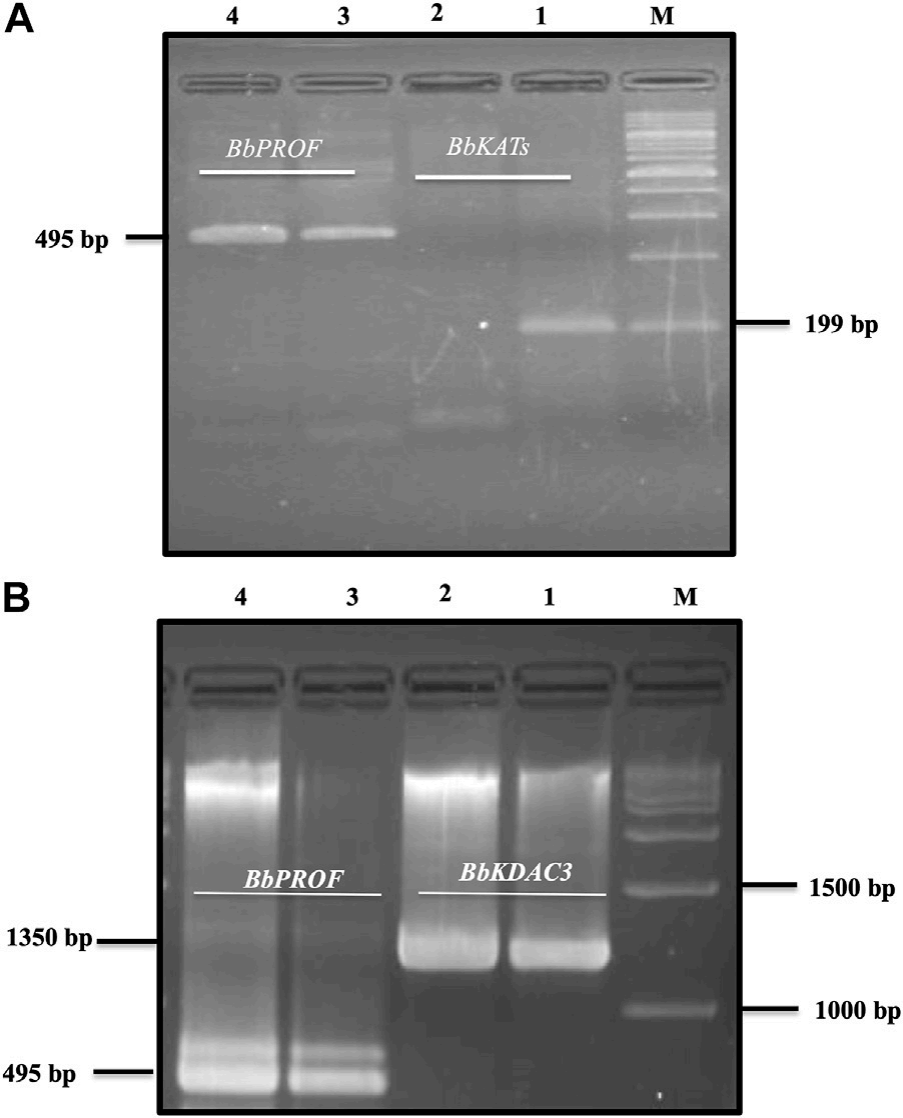
Reverse transcription-PCR analysis of *B. bovis* KATs (BbKATs), *B. bovis* KDAC3 (BbKDAC3) and *B. bovis* profilin (BbPROF) genes from *B. bovis* cultures treated with resveratrol at IC99 concentration and DMSO (0.1%) used as a control for 8 h **(A)**. BbKATs. **(B)**. BbKDAC3. Lanes 1 and 3 from the control culture; lanes 2 and 4 resveratrol-treated culture. M, molecular size marker.

Resveratrol at the IC99 concentration decreased the mRNA transcription of the target BbKATs but not the mRNA transcription of the control BbPROF gene within 8 h of treatment (Figure 3A). Furthermore, RVT treatment relatively promotes mRNA transcription of the target gene BbKDAC3 but does not inhibit mRNA transcription of the target gene BbKDAC3 but does not inhibit mRNA transcription of the control gene BbPROF (Figure 3B; Supplementary Figure S1).

### 3.3 RVT and ID a promising combination therapy for the treatment of babesiosis

**FIGURE 4:**
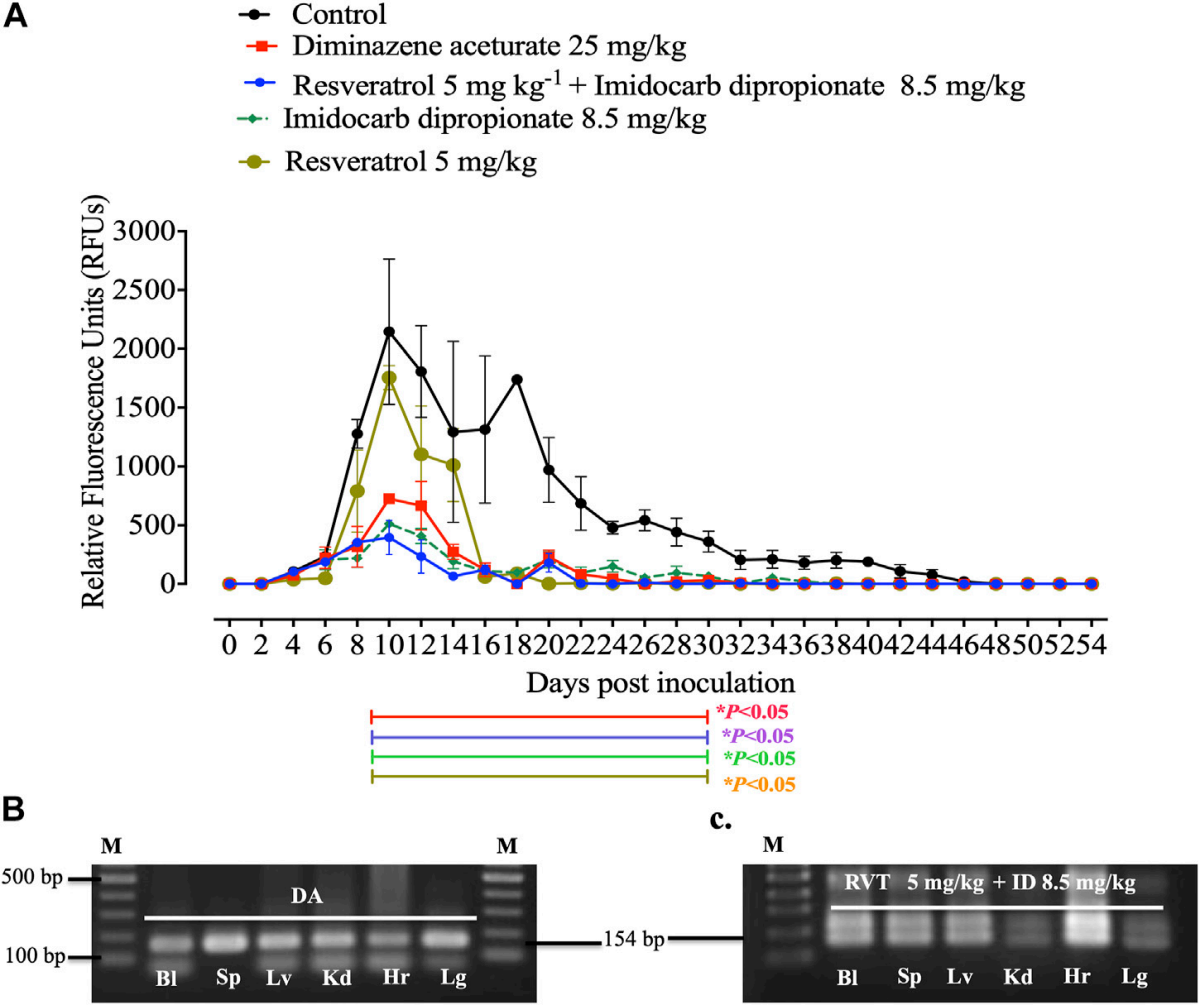
*In vivo* inhibitory efficacy of resveratrol. **(A)**. Inhibitory effect of resveratrol, imidocarb dipropionate and the combination of both drugs on the growth of Babesia microti. Each value represents the mean ± standard deviation of five mice per experimental group. Asterisks indicate significant differences (ANOVA; *p < 0.05) between the resveratrol-treated and control groups. **(B)**. PCR of the ss-rRNA gene in blood and different organs of **(B)**. microti-infected mice treated with 25 mg kg −1 diminazene aceturate (DA), and **(C)**. Imidocarb dipropionate (ID) combined with resveratrol (RVT). Bl, blood; Hr, heart; Lg, lung; Lv, liver; Kd, kidney; Sp, spleen. M indicates a 100 bp DNA ladder.

Unfortunately, there are no suitable laboratory experimental animals for bovine and equine Babesia infections. Alternatively, a mouse model infected with *B. Microti*, is commonly used for drug evaluation of the antibabesial drugs against animal babesiosis (Rizk et al., 2017a) since the inhibitory effect of the newly developed drug must be evaluated in laboratory animal to determine the possible side adverse effect of these hits before it is administration to animals under field condition. In the present study, RVT treatment alone displayed significant suppression (*p* < 0.05) in the emitted fluorescence signals from days 8–28 post-infection as compared with the positive control group (Figure 4A). Resveratrol showed an additive effect to the ID in combined treatment however, RVT showed an antagonistic effect to AZM at day 10 p. i. (Table 4). Peak fluorescence values in the treated groups with RVT 5 mg/kg and ID 8.5 mg/kg combinations reached an average of 395.70 at day 10 post-infection (pi). Meanwhile, fluorescence readings were significantly reduced (*p* < 0.05) in mice treated with RVT and ID combination from days 8–28 p. i. When compared to positive control mice (infected nontreated) (Figure 4A). Unfortunately, the parasite DNA was identified in the blood and other tissues of mice administered either DA monotherapy or RVT and ID combination based on nested PCR analysis (Figures 4B, C). Babesia microti DNA was identified in the blood and all analysed organs of infected mice treated with the solvent (positive control), however, no bands were found in non-treated non-infected mice (negative control) (Supplementary Figure S2). Although the present study determined the remnant of the parasite nucleic acid in the blood and other tissues of treated mice using nested PCR assay, further experiment as quantitative qPCR assay is necessary to determine the amount of nucleic acid in treated mice.

**TABLE 4:** Two drug interactions of resveratrol combined with either azithromycin or imidocarb dipropionate on the *in vivo* growth of *B. microti* on day 10 exhibited peak parasitemia.

Drug combination	FICD1	FICD2	ΣFIC	Degree of interaction^a^
**Azithromycin**				
	1	1.12	2.12	antagonistic
**Imidocarb dipropionate**				
	0.13	0.47	0.61	additive

^a^ The degree of drug interaction was determined based on the following fractional inhibitory concentration (FIC) index: >0.5–1 (additive), >1 to <2 (indifferent), and ≥2 (antagonistic). FICD1 is the fractional inhibitory concentration of resveratrol. Meanwhile, FICD2 is the fractional inhibitory concentration of either azithromycin or imidocarb dipropionate.

**FIGURE 5:**
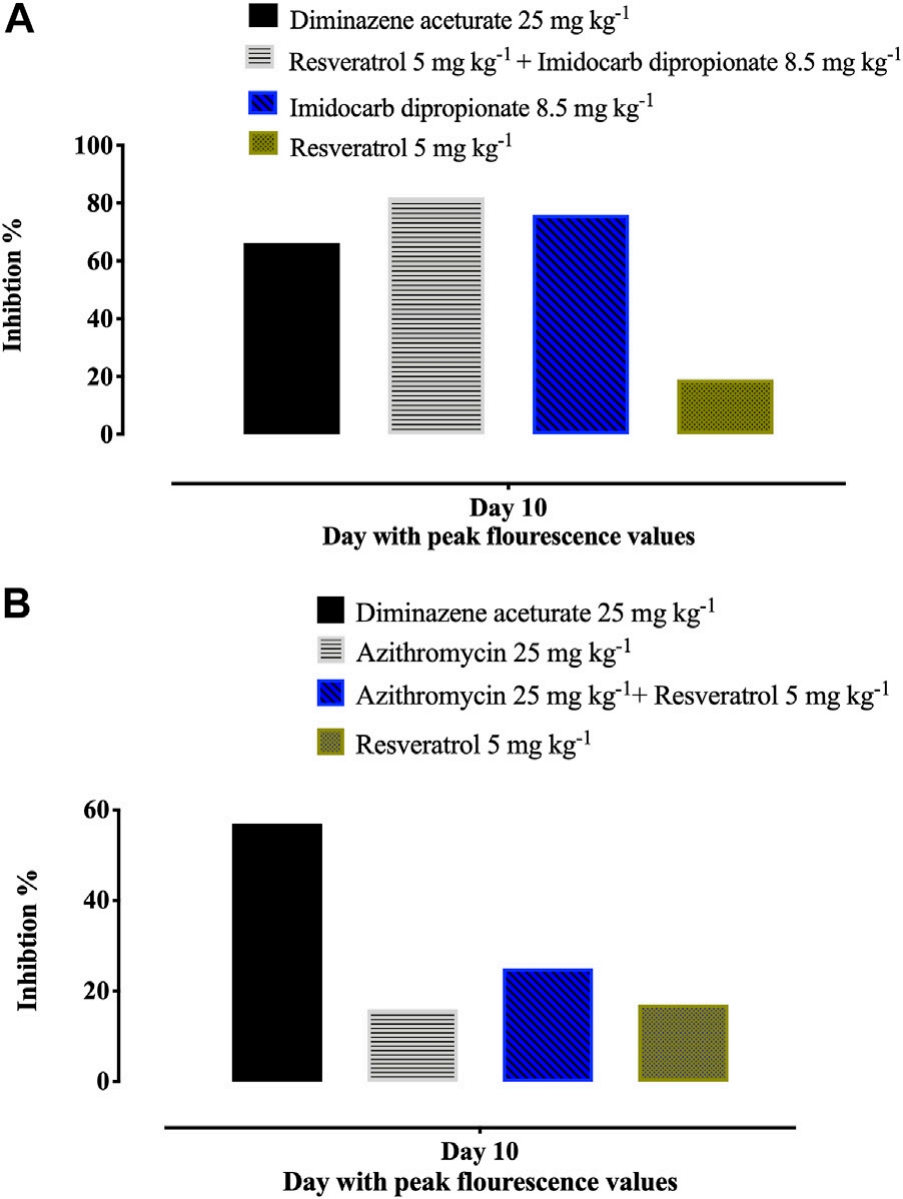
Percentages of inhibition in the growth of *B. Microti* in mice caused by resveratrol-mono-, and combination therapies on the days with peak parasitemia. **(A)** resveratrol and azithromycin. **(B)** resveratrol and imidocarb dipropionate. The % of parasite inhibition in each treated group was calculated as a ratio to the positive control group.

Notably, treatment with 5 mg/kg RVT+ 8.5 mg/kg ID resulted in 81.55% inhibition at day 10 p. i. (peak of parasitemia) as compared to 66.16% inhibition with 25 mg/kg DA and 76.13% inhibition with 8.5 mg/kg ID (Figure 5A). In addition, 5 mg/kg of RVT boosted the inhibition of *B. microti* by AZM (25 mg/kg) in mice (Figure 5B; Supplementary Figure S3).

### 3.4 RVT alleviated the cardiac side effect of AZM

Azithromycin has been suggested to have cardiotoxic effects thus to evaluate the ability of RVT to alleviate this side effect, cTnT levels in the plasma and heart tissues were evaluated by ELISA. At day 0, cTnT was nearly equal in the plasma of all mice groups, which decreased on day 10 (peak of parasitemia) to 99.25, 97.53, 101, and 98 pg/mL, and rise again to reach 110.61, 106.85, 105.18, and 106.73 pg/mL at day 28 post-infection in mice treated with RVT, RVT + AZM, positive control and non-treated mice, respectively (Supplementary Figure S4). Conversely, azithromycin treated group gradually increased plasma cTnT levels from day 0 (102.39 pg/mL) to 104.93 pg/mL at day 10 and 107.72 pg/mL at day 28 (Supplementary Figure S4).

**FIGURE 6:**
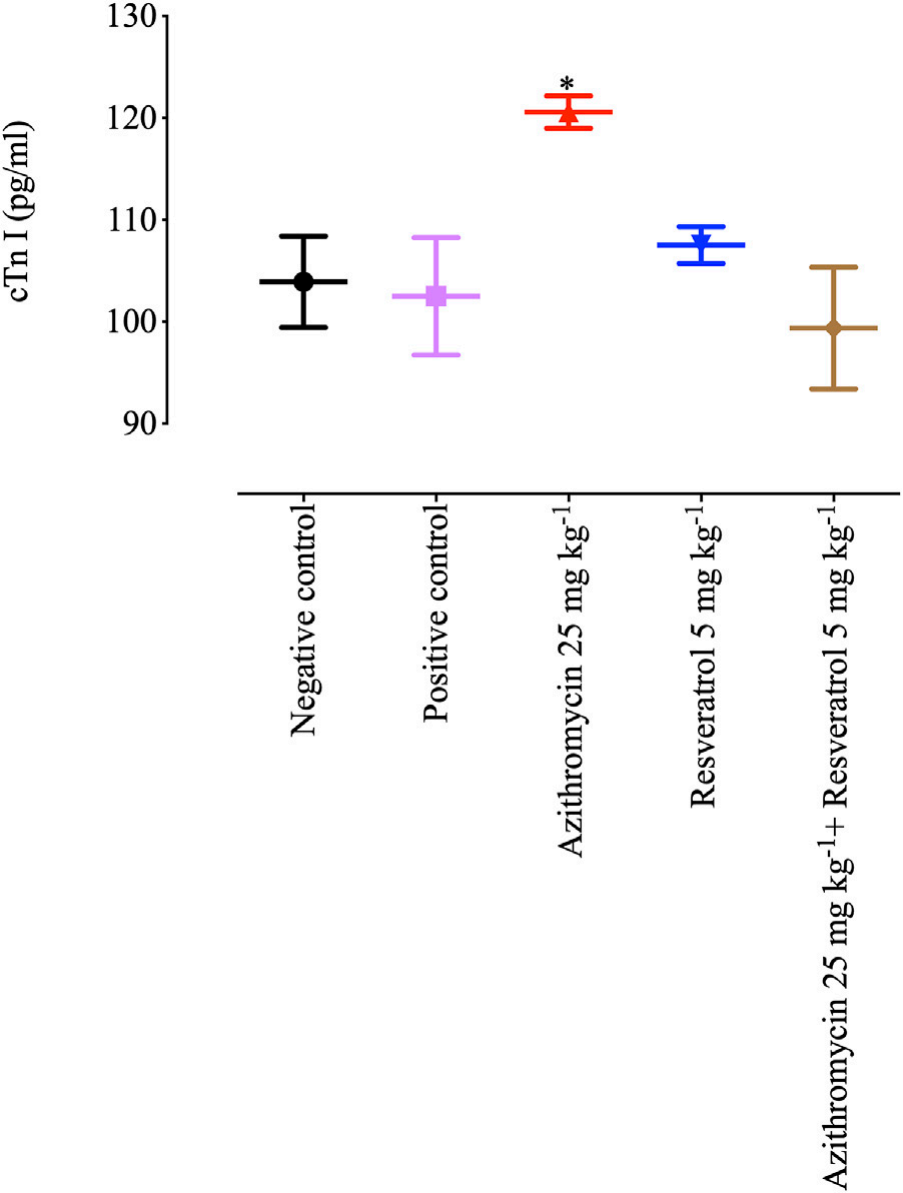
Cardiac troponin level in the heart tissues of mice treated with resveratrol and azithromycin combination therapy. Asterisks indicate significant differences (ANOVA; *p < 0.05) between the azithromycin-treated and other groups.

On day 28 post-infection, cTnT levels in heart tissue samples collected from *B. microti*–infected mice were significantly higher (*p* < 0.05) in the group treated with 25 mg/kg AZM (120.57 pg/mL) than any of the other groups (Figure 6). Of note, 5 mg/kg RVT significantly reduced (*p* < 0.05) cTnT level (99.35 pg/mL) in the heart tissue of treated mice as compared with those treated with 25 mg/kg AZM (Figure 6). These findings suggest the possible role of RVT to alleviate the cardiotoxic effects of AZM.

## 4 Discussion

The unique metabolic characteristic of Babesia makes the development of anti-babesial drugs a difficult issue. Various compounds targeting epigenetic enzymes that regulate histone modification to activate or silence gene expression have been discovered for the treatment of several diseases (Brownell et al., 1996; Berman et al., 2017). Importantly, histone methyltransferases and histone deacetylase inhibitors have been investigated as possible targets for the development of novel drugs in several protozoan diseases (Zuma and de Souza, 2018). Resveratrol has attracted much attention for its ability to enhance the deacetylase activity of protein deacetylase sirtuin 1 (Sirt1) which is a NAD-dependent histone deacetylase (Yu et al., 2009). Sirt1 is a major regulator of multiple biological processes, including the stress response, apoptosis, the regulation of gene transcription and the cell cycle (Michan and Sinclair, 2007). In addition, a resveratrol-mediated increase in Sirt1 led to cell death in HaCaT keratinocytes (Lee et al., 2016). Moreover, RVT KDACs activation effect has been confirmed on a parasitic protozoan, T. cruzi because it suppresses T. cruzi epimastigote growth and infectivity of cell-derived trypomastigotes, as well as intracellular amastigote differentiation and/or replication by activation of KDACs III (Campo, 2017; Rodriguez et al., 2022). Subsequently, the present study investigated RVT as an inhibitor of KATs and an activator of KDAC for the potential activity against piroplasmosis and to offer an alternative strategy to discover a promising compound against such infectious disease. Although, further future studies are required to confirm such effect of RVT on KATs and KDAC using a quantitative real time-PCR assay.

Resveratrol exhibited a potent inhibitory effect against *B. bovis in vitro*. Furthermore, the obtained *in vivo* results suggest that RVT + ID could be a viable combination therapy to overcome the side effects and drug residues in tissues attributed to the full doses of the widely used antibabesial drug in the field, ID. Thus, different theories are suggested for the antiprotozoal activities of RVT. For example, it inhibited the virulence of E. histolytica trophozoites *in vitro* and *in vivo* by arresting cell development and causing oxidative stress, which eventually produced apoptosis-like trophozoites (Pais-Morales et al., 2016). Resveratrol also demonstrated strong antileishmanial efficacy against Leishmania major and Leishmania amazonensis (Mousavi et al., 2022). Resveratrol’s trypanocidal activity has been linked to its ability to inhibit the arginine kinase of T. cruzi (Valera Vera et al., 2016). RVT reduced oxidative damage and prevent the behavioural changes seen in Toxoplasma gondii (T. gondii)-infected mice (Bottari et al., 2015a; Bottari et al., 2015b; Bottari et al., 2016). Resveratrol successfully blocks the redox homeostasis of RH tachyzoites, alleviates host macrophage stress brought on by parasite infection and eventually aids macrophages in eliminating intracellular tachyzoites by affecting histone acetylation levels and could be generating DNA damage (Chen et al., 2019; Contreras et al., 2021). In addition, resveratrol was proposed for congenital toxoplasmosis therapy because it increased cell proliferation, restored cellular viability of infected neural progenitor cells and inhibited gliogenesis of infected neural progenitor cells, favouring neuronal maturation (Bottari et al., 2019).

Resveratrol treatment activates transcription of the BbKDAC3 gene while inhibiting transcription of the BbKATs as indicated by RT-PCR. These findings indicate that RVT has direct antipiroplasm properties. Moreover, resveratrol stimulation of BbKDAC3 is linked to the direct activation of sirt1, and a histone deacetylase (KDAC) that deacetylates various histone and non-histone proteins (Milne and Denu, 2008). On the one hand, sirt1 also shuttles between the nucleus and the cytoplasm and is highly expressed in endothelial cells (Li and Forstermann, 2009). On the other hand, it was thought that RVT-sirt1 interaction demonstrated resveratrol’s regulatory impact on cardiovascular diseases (Ministrini et al., 2021). Sirt1 stimulates nitric oxide synthase (eNOS) gene transcription and mRNA stability, thereby resulting in endothelial relaxation, a mechanism that is always compromised to some degree in patients with different cardiovascular diseases (Ministrini et al., 2021). Thus, herein, the effect of RVT on cardiac toxicity associated with treatment with AZM is studied to investigate these proprieties. AZM increases the risk of cardiovascular death in persons with baseline risk factors for abnormal heart rhythms (Mortensen et al., 2014; Rao et al., 2014; Lu et al., 2015). However, under these conditions, the negative effects of this drug remain present, but they are the only available therapy choices for human babesiosis. Therefore, looking for drugs that reduce their adverse effects would be beneficial. Our findings suggest that RVT can reduce the cardiotoxic effect of AZM on mice during *B. Microti* treatment, as measured by cTnT level in heart tissues. The troponin complex plays a significant role in regulating skeletal and cardiac muscle contraction (Gomes et al., 2002; Parmacek and Solaro, 2004). The binary IC complex and free troponin T are the two main forms of the troponin complex that are released into the bloodstream gradually after an incident such as a myocardial infarction (Olkowicz et al., 2015). Troponins (particularly troponin T) are recognised as effective biomarkers for diagnosing myocardial injury, with greater specificity and timing of the release as compared to cardiac isoforms of aspartate transaminase (AST), creatine kinase (CK) and lactate dehydrogenase (LDH) (Lewandrowski et al., 2002; Olkowicz et al., 2015). The treatment of mice infected with T. cruzi with RVT (a natural SIRT1 agonist) increases the number of mitochondria as well as the expression of genes for oxidative phosphorylation, thereby resulting in a partial recovery of cardiac output and better control of left ventricular mass in infected mice, as well as a decrease in myocardial inflammation (Wan et al., 2016). Such findings indicate that RVT may have a role in reducing the cardiotoxic effects of AZM and closely related drugs.

## 5 Conclusion

Resveratrol inhibits the growth of bovine and equine piroplasms *in vitro*, with *B. bovis* being the most sensitive treated parasite. The combined treatment consisting of RVT and ID is more effective for *B. Microti-infected mice* than the currently used antibabesial monotherapies. Thus, resveratrol may play a protective function in reducing the cardiotoxic effects of AZM and closely related medications. The molecular mechanisms underlying anti-piroplasms and cardioprotective activities of RVT may be attributed to the ability of resveratrol to stimulate KDAC through activation of SIRT 1. However, further studies are necessary to explore such mechanisms in piroplasm.

## Data Availability

The original contributions presented in the study are included in the article/Supplementary Material, further inquiries can be directed to the corresponding authors.

## Supplementary material

SUPPLEMENTARY FIGURE S1 RT-PCR image gel analysis of *B. Bovis* KDAC3 (*BbKDAC3*) gene from cultures treated with resveratrol at IC99 concentration and DMSO (0.1%) used as a control for 8 h, **(A)** control culture, **(B)** Resveratrol treated culture. The analysis was performed using Image J software.

SUPPLEMENTARY FIGURE S2 PCR of the *ss-rRNA* gene in blood and different organs of *B. Microti*-infected mice treated with the solvent (positive control), and non-treated mice (negative control). PC, positive control; NC, negative control; Bl, blood; Hr, heart; Lg, lung; Lv, liver; Kd, kidney; Sp, spleen. M indicates a 100 bp DNA ladder.

SUPPLEMENTARY FIGURE S3 Inhibitory effect of resveratrol, azithromycin, and the combination of both drugs on the growth of *Babesia microti*. Each value represents the mean ± standard deviation of five mice per experimental group.

SUPPLEMENTARY FIGURE S4 Cardiac troponin level in the plasma of mice treated with resveratrol and azithromycin combination therapy.

## References

[B1] Aboulaila M. Munkhjargal T. Sivakumar T. Ueno A. Nakano Y. Yokoyama M. (2012). Apicoplast-targeting antibacterials inhibit the growth of Babesia parasites. Antimicrob. Agents Chemother. 56, 3196–3206. 10.1128/AAC.05488-11 22391527 PMC3370714

[B2] Aggarwal B. B. Bhardwaj A. Aggarwal R. S. Seeram N. P. Shishodia S. Takada Y. (2004). Role of resveratrol in prevention and therapy of cancer: Preclinical and clinical studies. Anticancer Res. 24, 2783–2840. 15517885

[B3] Berman A. Y. Motechin R. A. Wiesenfeld M. Y. Holz M. K. (2017). The therapeutic potential of resveratrol: A review of clinical trials. NPJ Precis. Oncol. 1. 10.1038/s41698-017-0038-6 PMC5630227 28989978

[B4] Bhat K. P. L. Kosmeder J. W. Pezzuto J. M. Pezzuto J. M. (2001). Biological effects of resveratrol. Antioxidants Redox Signal. 3, 1041–1064. 10.1089/152308601317203567 11813979

[B5] Bostanghadiri N. Pormohammad A. Chirani A. S. Pouriran R. Erfanimanesh S. Hashemi A. (2017). Comprehensive review on the antimicrobial potency of the plant polyphenol Resveratrol. Biomed. Pharmacother. 95, 1588–1595. 10.1016/j.biopha.2017.09.084 28950659

[B6] Bottari N. B. Baldissera M. D. Tonin A. A. Rech V. C. Alves C. B. D'Avila F. (2016). Synergistic effects of resveratrol (free and inclusion complex) and sulfamethoxazole-trimetropim treatment on pathology, oxidant/antioxidant status and behavior of mice infected with Toxoplasma gondii. Microb. Pathog. 95, 166–174. 10.1016/j.micpath.2016.04.002 27057672

[B7] Bottari N. B. Baldissera M. D. Tonin A. A. Rech V. C. Nishihira V. S. Thomé G. R. (2015a). Effects of sulfamethoxazole-trimethoprim associated to resveratrol on its free form and complexed with 2-hydroxypropyl-β-cyclodextrin on cytokines levels of mice infected by Toxoplasma gondii. Microb. Pathog. 87, 40–44. 10.1016/j.micpath.2015.07.013 26209515

[B8] Bottari N. B. Baldissera M. D. Tonin A. A. Rech V. C. Nishihira V. S. Thomé G. R. (2015b). Sulfamethoxazole-trimethoprim associated with resveratrol for the treatment of toxoplasmosis in mice: Influence on the activity of enzymes involved in brain neurotransmission. Microb. Pathog. 79, 17–23. 10.1016/j.micpath.2015.01.001 25572158

[B9] Bottari N. B. Schetinger M. R. C. Pillat M. M. Palma T. V. Ulrich H. Alves M. S. (2019). Resveratrol as a therapy to restore neurogliogenesis of neural progenitor cells infected by Toxoplasma gondii. Mol. Neurobiol. 56, 2328–2338. 10.1007/s12035-018-1180-z 30027338

[B10] Brownell J. E. Zhou J. Ranalli T. Kobayashi R. Edmondson D. G. Roth S. Y. (1996). Tetrahymena histone acetyltransferase A: A homolog to yeast Gcn5p linking histone acetylation to gene activation. Cell 84, 843–851. 10.1016/s0092-8674(00)81063-6 8601308

[B11] Campo V. A. (2017). Comparative effects of histone deacetylases inhibitors and resveratrol on Trypanosoma cruzi replication, differentiation, infectivity and gene expression. Int. J. Parasitol. Drugs Drug Resist 7, 23–33. 10.1016/j.ijpddr.2016.12.003 28038431 PMC5199159

[B12] Chen Q. W. Dong K. Qin H. X. Yang Y. K. He J. L. Li J. (2019). Direct and indirect inhibition effects of resveratrol against Toxoplasma gondii tachyzoites *in vitro* . Antimicrob. Agents Chemother. 63. 10.1128/AAC.01233-18 PMC6395936 30530601

[B13] Contreras S. M. Ganuza A. Corvi M. M. Angel S. O. (2021). Resveratrol induces H3 and H4K16 deacetylation and H2A.X phosphorylation in Toxoplasma gondii. BMC Res. Notes 14, 19. 10.1186/s13104-020-05416-4 33413578 PMC7792170

[B14] Croken M. M. Nardelli S. C. Kim K. (2012). Chromatin modifications, epigenetics, and how protozoan parasites regulate their lives. Trends Parasitol. 28, 202–213. 10.1016/j.pt.2012.02.009 22480826 PMC3340475

[B15] Duarte A. Martinho A. Luís Â. Figueiras A. Oleastro M. Domingues F. C. (2015). Resveratrol encapsulation with methyl-β-cyclodextrin for antibacterial and antioxidant delivery applications. LWT - Food Sci. Technol. 63, 1254–1260. 10.1016/j.lwt.2015.04.004

[B16] Elsworth B. Duraisingh M. T. (2021). A framework for signaling throughout the life cycle of Babesia species. Mol. Microbiol. 115, 882–890. 10.1111/mmi.14650 33274587 PMC10209834

[B17] Ferreira C. Soares D. C. Nascimento M. T. Pinto-da-Silva L. H. Sarzedas C. G. Tinoco L. W. (2014). Resveratrol is active against Leishmania amazonensis: *In vitro* effect of its association with amphotericin B. Antimicrob. Agents Chemother. 58, 6197–6208. 10.1128/AAC.00093-14 25114129 PMC4187957

[B18] Gibney E. R. Nolan C. M. (2010). Epigenetics and gene expression. Hered. (Edinb) 105, 4–13. 10.1038/hdy.2010.54 20461105

[B19] Gomes A. V. Potter J. D. Szczesna-Cordary D. (2002). The role of troponins in muscle contraction. IUBMB Life 54, 323–333. 10.1080/15216540216037 12665242

[B20] Gurboga M. Kugu G. Kamberaj H. Mutlu O. (2021). Identification of benzamide inhibitors of histone deacetylase 1 from Babesia and Theileria species via high-throughput virtual screening and molecular dynamics simulations. Parasitol. Res. 120, 2175–2187. 10.1007/s00436-021-07158-z 33987736

[B21] Langcake P. C. Pryce R. J. (1979). Identification of pterostilbene as a phytoalexin from Vitis vinifera leaves. Phytochemistry 18, 1025–1027. 10.1016/s0031-9422(00)91470-5

[B22] Lee J. H. Kim J. S. Park S. Y. Lee Y. J. (2016). Resveratrol induces human keratinocyte damage via the activation of class III histone deacetylase, Sirt1. Oncol. Rep. 35, 524–529. 10.3892/or.2015.4332 26499368

[B23] Lewandrowski K. Chen A. Januzzi J. (2002). Cardiac markers for myocardial infarction. A brief review. Am. J. Clin. Pathol. 118, S93–S99. 10.1309/3EK7-YVV9-228C-E1XT 14569816

[B24] Li H. Förstermann U. (2009). Resveratrol: A multifunctional compound improving endothelial function. Editorial to: "Resveratrol supplementation gender independently improves endothelial reactivity and suppresses superoxide production in healthy rats" by S. Soylemez et al. Cardiovasc Drugs Ther. 23, 425–429. 10.1007/s10557-009-6209-0 19937102 PMC2797420

[B25] Li X W. J. Wang Y. Nian Y. Zhao S. Liu J. Luo J. (2022). Histone methyltransferases inhibitors againstBabesia *in vitro* .

[B26] Lu Z. K. Yuan J. Li M. Sutton S. S. Rao G. A. Jacob S. (2015). Cardiac risks associated with antibiotics: Azithromycin and levofloxacin. Expert Opin. Drug Saf. 14, 295–303. 10.1517/14740338.2015.989210 25494485 PMC4404501

[B27] Michan S. Sinclair D. (2007). Sirtuins in mammals: Insights into their biological function. Biochem. J. 404, 1–13. 10.1042/BJ20070140 17447894 PMC2753453

[B28] Milne J. C. Denu J. M. (2008). The sirtuin family: Therapeutic targets to treat diseases of aging. Curr. Opin. Chem. Biol. 12, 11–17. 10.1016/j.cbpa.2008.01.019 18282481

[B29] Ministrini S. Puspitasari Y. M. Beer G. Liberale L. Montecucco F. Camici G. G. (2021). Sirtuin 1 in endothelial dysfunction and cardiovascular aging. Front. Physiol. 12, 733696. 10.3389/fphys.2021.733696 34690807 PMC8527036

[B30] Mortensen E. M. Halm E. A. Pugh M. J. Copeland L. A. Metersky M. Fine M. J. (2014). Association of azithromycin with mortality and cardiovascular events among older patients hospitalized with pneumonia. Jama 311, 2199–2208. 10.1001/jama.2014.4304 24893087 PMC4109266

[B31] Mousavi P. Rahimi Esboei B. Pourhajibagher M. Fakhar M. Shahmoradi Z. Hejazi S. H. (2022). Anti-leishmanial effects of resveratrol and resveratrol nanoemulsion on Leishmania major. BMC Microbiol. 22, 56. 10.1186/s12866-022-02455-8 35168553 PMC8845381

[B32] Munkhjargal T. Aboulaila M. R. A. Sivakumar T. Yokoyama N. Igarashi I. (2009). Inhibitory effect of apicidin on *in vitro* and *in vivo* growth of Babesia parasites. J. Protozool. Res. 19, 42–49.

[B33] Munkhjargal T. Aboulaila M. Ueno A. Sivakumar T. Nakano Y. Yokoyama M. (2012). Cloning and characterization of histone deacetylase from Babesia bovis. Vet. Parasitol. 190, 423–433. 10.1016/j.vetpar.2012.06.026 22818786

[B34] Olkowicz M. Rybakowska I. Chlopicki S. Smolenski R. T. (2015). Development and analytical comparison of microflow and nanoflow liquid chromatography/mass spectrometry procedures for quantification of cardiac troponin T in mouse hearts. Talanta 131, 510–520. 10.1016/j.talanta.2014.08.029 25281134

[B35] Pais-Morales J. Betanzos A. García-Rivera G. Chávez-Munguía B. Shibayama M. Orozco E. (2016). Resveratrol induces apoptosis-like death and prevents *in vitro* and *in vivo* virulence of Entamoeba histolytica. PLoS One 11, e0146287. 10.1371/journal.pone.0146287 26731663 PMC4701480

[B36] Parmacek M. S. Solaro R. J. (2004). Biology of the troponin complex in cardiac myocytes. Prog. Cardiovasc Dis. 47, 159–176. 10.1016/j.pcad.2004.07.003 15736582

[B37] Rao G. A. Mann J. R. Shoaibi A. Bennett C. L. Nahhas G. Sutton S. S. (2014). Azithromycin and levofloxacin use and increased risk of cardiac arrhythmia and death. Ann. Fam. Med. 12, 121–127. 10.1370/afm.1601 24615307 PMC3948758

[B38] Rizk M. A. El-Sayed S. A. AbouLaila M. Tuvshintulga B. Yokoyama N. Igarashi I. (2016). Large-scale drug screening against Babesia divergens parasite using a fluorescence-based high-throughput screening assay. Vet. Parasitol. 227, 93–97. 10.1016/j.vetpar.2016.07.032 27523944

[B39] Rizk M. A. El-Sayed S. a. E. AbouLaila M. Eltaysh R. Yokoyama N. Igarashi I. (2017a). Performance and consistency of a fluorescence-based high-throughput screening assay for use in Babesia drug screening in mice. Sci. Rep. 7, 12774. 10.1038/s41598-017-13052-5 29038534 PMC5643553

[B40] Rizk M. A. El-Sayed S. a. E. AbouLaila M. Yokoyama N. Igarashi I. (2017b). Evaluation of the inhibitory effect of N-acetyl-L-cysteine on Babesia and Theileria parasites. Exp. Parasitol. 179, 43–48. 10.1016/j.exppara.2017.06.003 28655583

[B41] Rizk M. A. El-Sayed S. a. E. Igarashi I. (2021). Evaluation of the inhibitory effect of Zingiber officinale rhizome on Babesia and Theileria parasites. Parasitol. Int. 85, 102431. 10.1016/j.parint.2021.102431 34352378

[B42] Rizk M. A. El-Sayed S. A. Terkawi M. A. Youssef M. A. El Said S. Elsayed G. (2015). Optimization of a fluorescence-based assay for large-scale drug screening against Babesia and Theileria parasites. PLoS One 10, e0125276. 10.1371/journal.pone.0125276 25915529 PMC4411034

[B43] Rizk M. A. El-Sayed S. E. Nassif M. Mosqueda J. Xuan X. Igarashi I. (2020). Assay methods for *in vitro* and *in vivo* anti-Babesia drug efficacy testing: Current progress, outlook, and challenges. Vet. Parasitol. 279, 109013. 10.1016/j.vetpar.2019.109013 32070899

[B44] Rizk M. A. El-Sayed S. Igarashi I. (2023). Diminazene aceturate and imidocarb dipropionate-based combination therapy for babesiosis - a new paradigm. Ticks Tick. Borne Dis. 14, 102145. 10.1016/j.ttbdis.2023.102145 37011497

[B45] Rodriguez M. E. Tekiel V. Campo V. A. (2022). *In vitro* evaluation of Resveratrol as a potential pre-exposure prophylactic drug against Trypanosoma cruzi infection. Int. J. Parasitol. Drugs Drug Resist 20, 54–64. 10.1016/j.ijpddr.2022.08.003 36099853 PMC9474288

[B46] Sindikubwabo F. Ding S. Hussain T. Ortet P. Barakat M. Baumgarten S. (2017). Modifications at K31 on the lateral surface of histone H4 contribute to genome structure and expression in apicomplexan parasites. Elife 6. 10.7554/eLife.29391 PMC5685513 29101771

[B47] Takaoka M. (1939). The phenolic substances of white hellebore (Veratrum grandiflorum loes fil) II. Nippon. Kagaku Kaishi 60, 1261–1264. 10.1246/nikkashi1921.60.1261

[B48] Valera Vera E. A. Sayé M. Reigada C. Damasceno F. S. Silber A. M. Miranda M. R. (2016). Resveratrol inhibits Trypanosoma cruzi arginine kinase and exerts a trypanocidal activity. Int. J. Biol. Macromol. 87, 498–503. 10.1016/j.ijbiomac.2016.03.014 26976067

[B49] Vanagas L. Jeffers V. Bogado S. S. Dalmasso M. C. Sullivan W. J. Jr. Angel S. O. (2012). Toxoplasma histone acetylation remodelers as novel drug targets. Expert Rev. Anti Infect. Ther. 10, 1189–1201. 10.1586/eri.12.100 23199404 PMC3581047

[B50] Wan X. Wen J. J. Koo S. J. Liang L. Y. Garg N. J. (2016). SIRT1-PGC1α-NFκB pathway of oxidative and inflammatory stress during trypanosoma cruzi infection: Benefits of SIRT1-targeted therapy in improving heart function in chagas disease. PLoS Pathog. 12, e1005954. 10.1371/journal.ppat.1005954 27764247 PMC5072651

[B51] Wapenaar H. Dekker F. J. (2016). Histone acetyltransferases: Challenges in targeting bi-substrate enzymes. Clin. Epigenetics 8, 59. 10.1186/s13148-016-0225-2 27231488 PMC4881052

[B52] Yu W. Fu Y. C. Zhou X. H. Chen C. J. Wang X. Lin R. B. (2009). Effects of resveratrol on H(2)O(2)-induced apoptosis and expression of SIRTs in H9c2 cells. J. Cell Biochem. 107, 741–747. 10.1002/jcb.22169 19415680

[B53] Zuma A. A. de Souza W. (2018). Histone deacetylases as targets for antitrypanosomal drugs. Future Sci. OA 4, Fso325. 10.4155/fsoa-2018-0037 30271613 PMC6153458

