# Supplementary material for: Evaluating the inhibitory effect of resveratrol on the multiplication of several Babesia species and Theileria equi on in vitro cultures, and Babesia microti in mice
Source: Front Pharmacol. 2023 May 30;14:1192999. doi: 10.3389/fphar.2023.1192999 (PMC10267976; doi:10.3389/fphar.2023.1192999)
Supplement: Supplementary file 1 [file Table1.pdf]

Table S1
Viability test results of resveratrol drug evaluated for *Babesia* and *Theileria* parasite

**Drug**	**Drug concentrations (μM)** [a]											
	0.005	0.05	0.12	0.25	0.5	1	5	10	25	50	100	200
B. bovis	+	+	+	+	+	+	+	+	+	−	−	−
B. bigemina	+	+	+	+	+	+	+	+	+	+	+	−
B. divergens	+	+	+	+	+	+	+	+	+	+	+	−
T. equi	+	+	+	+	+	+	+	+	+	+	+	−
B. caballi	+	+	+	+	+	+	+	+	+	+	+	−

a Each value was calculated in three separate experiments. Each concentration of the drug was made in triplicate in each experiment. + = viable; − = dead
