# Supplementary figures and images for: Evaluating the inhibitory effect of resveratrol on the multiplication of several Babesia species and Theileria equi on in vitro cultures, and Babesia microti in mice
Source: Front Pharmacol. 2023 May 30;14:1192999. doi: 10.3389/fphar.2023.1192999 (PMC10267976; doi:10.3389/fphar.2023.1192999)

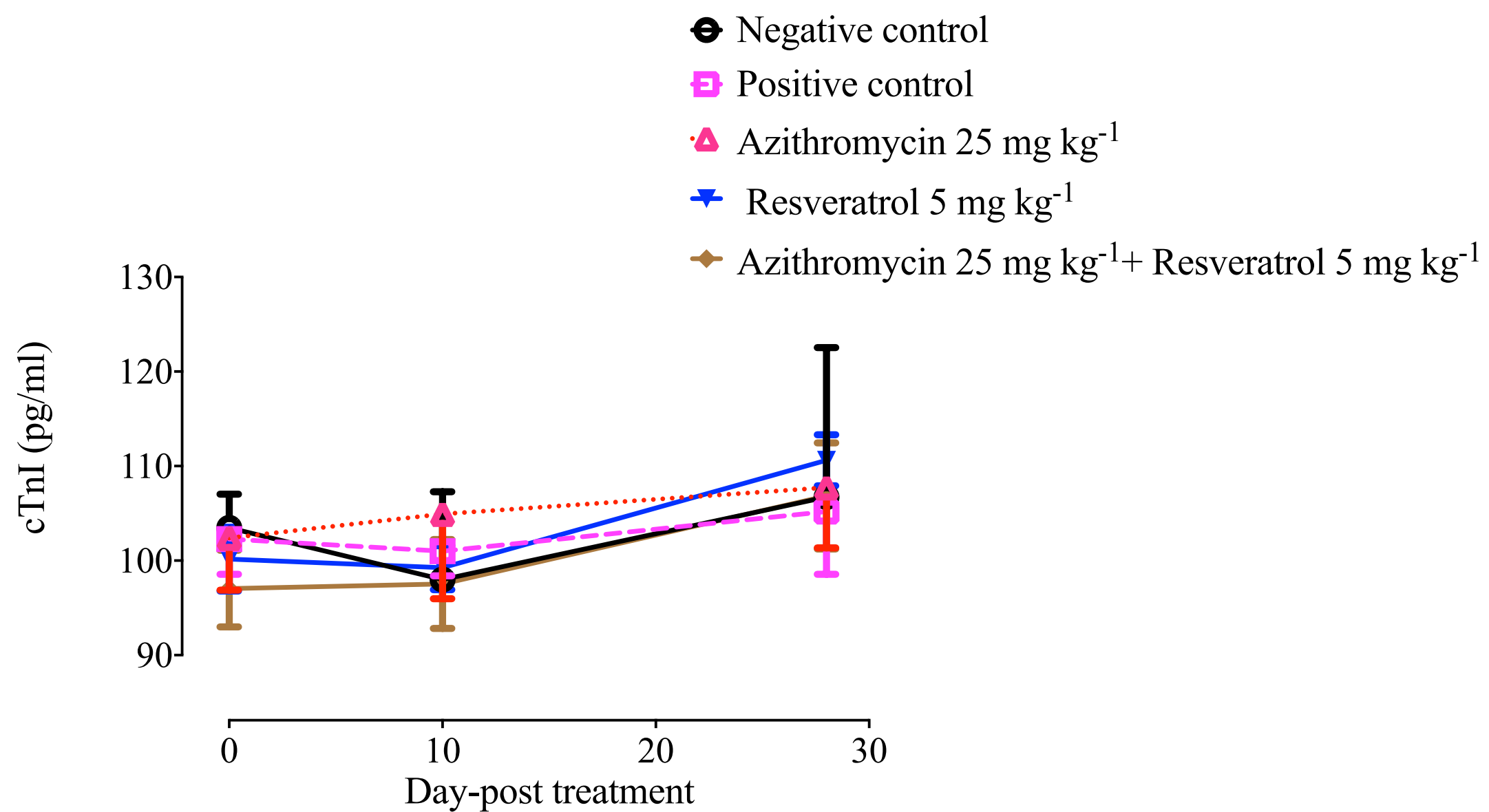

Supplement: Supplementary file 2 [file Image4.pdf]

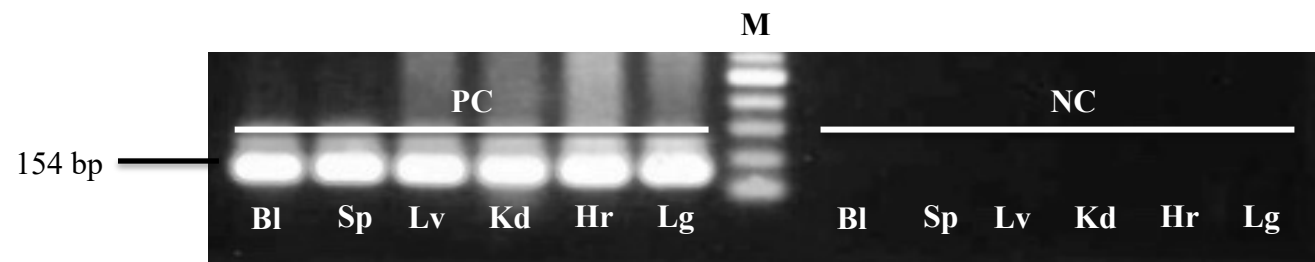

Supplement: Supplementary file 3 [file Image2.pdf]

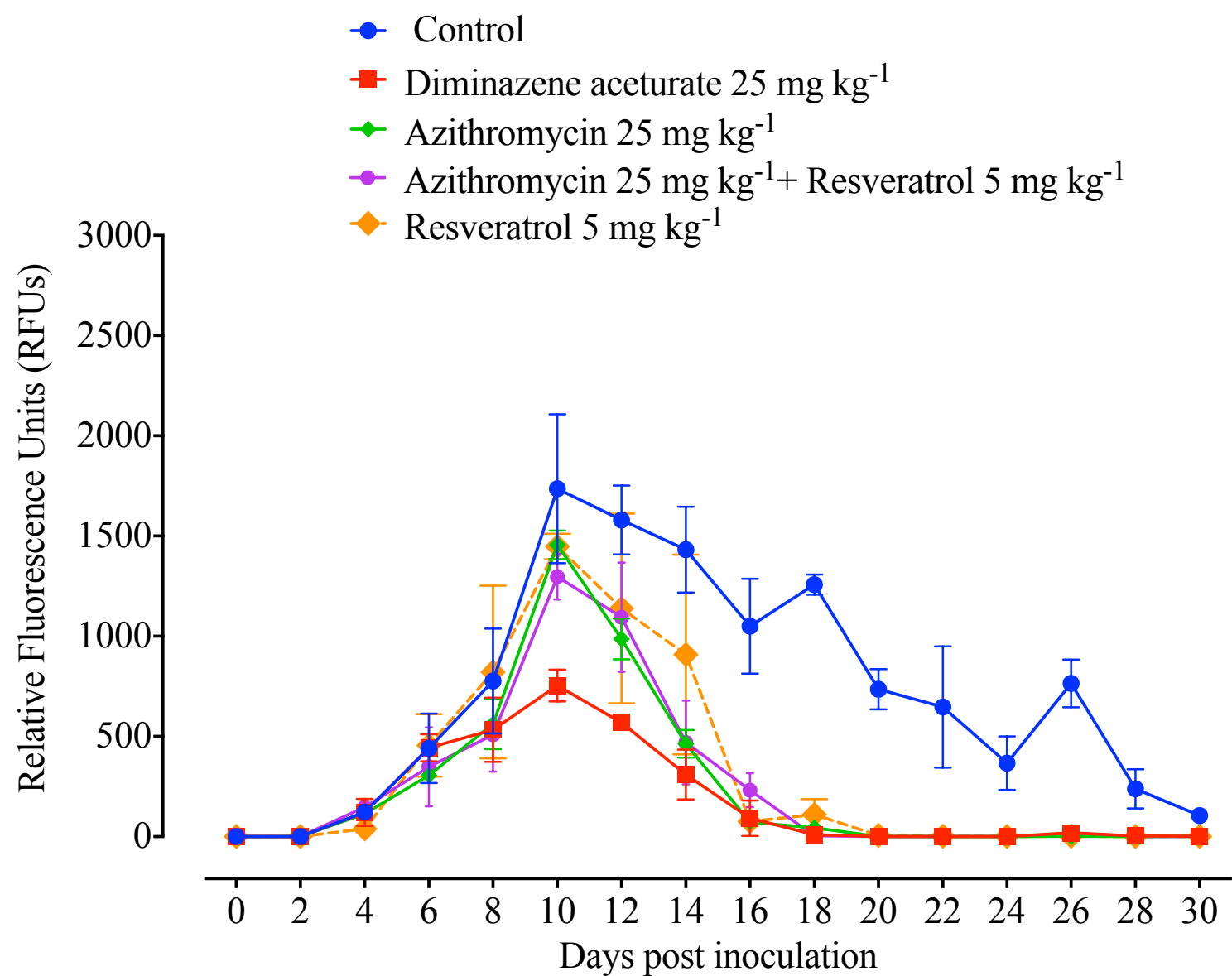

Supplement: Supplementary file 4 [file Image3.pdf]

(a)

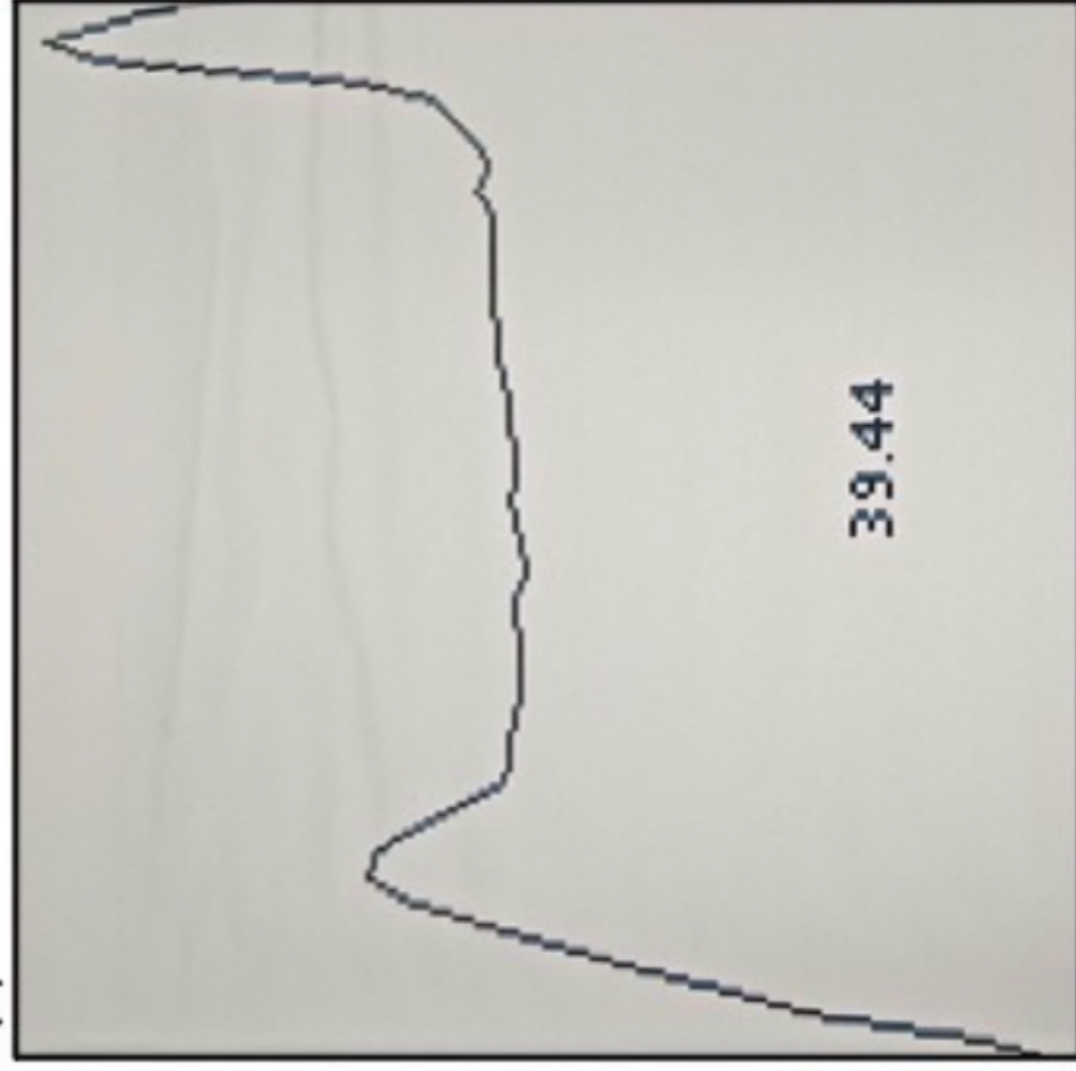

(b)

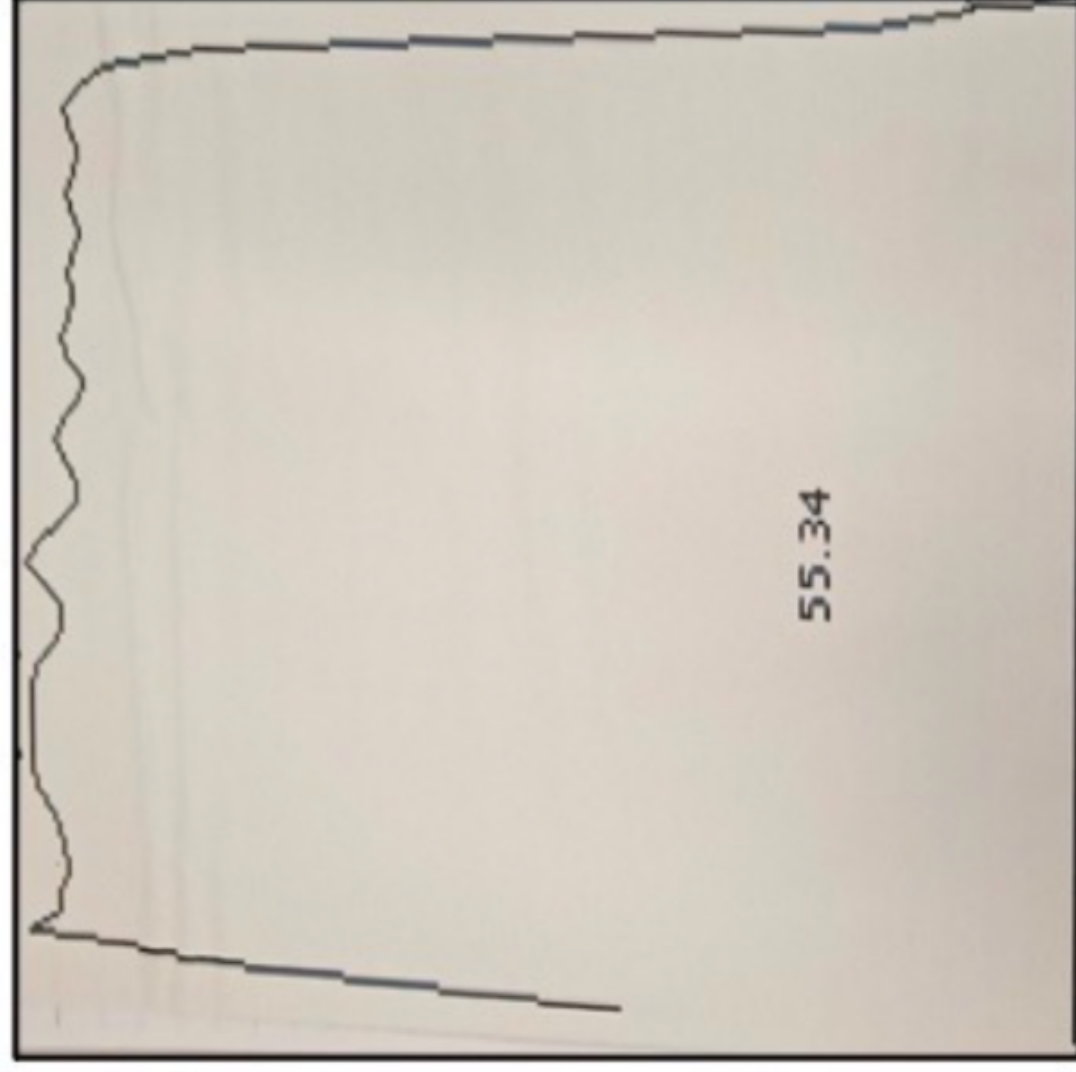

Supplement: Supplementary file 5 [file Image1.pdf]
